# Hereditary lissencephaly and cerebellar hypoplasia in Churra lambs

**DOI:** 10.1186/1746-6148-9-156

**Published:** 2013-08-09

**Authors:** Valentín Pérez, Aroa Suárez-Vega, Miguel Fuertes, Julio Benavides, Laetitia Delgado, M Carmen Ferreras, Juan José Arranz

**Affiliations:** 1 Departamento de Sanidad Animal (Anatomía Patológica), Instituto de Ganadería de Montaña (CSIC-ULE), Facultad de Veterinaria, Universidad de León, Campus de Vegazana s/n, León 24071, Spain; 2 Departamento de Producción Animal, Facultad de Veterinaria, Universidad de León, Campus de Vegazana s/n, León 24071, Spain

**Keywords:** Lissencephaly, Cerebellar hypoplasia, Agyria-pachygyria, Sheep, Autosomal recessive

## Abstract

**Background:**

Lissencephaly is a rare developmental brain disorder in veterinary and human medicine associated with defects in neuronal migration leading to a characteristic marked reduction or absence of the convolutional pattern of the cerebral hemispheres. In many human cases the disease has a genetic basis. In sheep, brain malformations, mainly cerebellar hypoplasia and forms of hydrocephalus, are frequently due to *in utero* viral infections. Although breed-related malformations of the brain have been described in sheep, breed-related lissencephaly has not been previously recorded in a peer reviewed publication.

**Results:**

Here we report neuropathological findings in 42 newborn lambs from a pure Churra breed flock, with clinical signs of weakness, inability to walk, difficulty in sucking and muscular rigidity observed immediately after birth. All the lambs showed near-total agyria with only a rudimentary formation of few sulci and gyri, and a severe cerebellar hypoplasia. On coronal section, the cerebral grey matter was markedly thicker than that of age-matched unaffected lambs and the ventricular system was moderately dilated. Histologically, the normal layers of the cerebral cortex were disorganized and, using an immunohistochemical technique against neurofilaments, three layers were identified instead of the six present in normal brains. The hippocampus was also markedly disorganised and the number and size of lobules were reduced in the cerebellum. Heterotopic neurons were present in different areas of the white matter. The remainder of the brain structures appeared normal. The pathological features reported are consistent with the type LCH-b (lissencephaly with cerebellar hypoplasia group b) defined in human medicine. No involvement of pestivirus or bluetongue virus was detected by immunohistochemistry. An analysis of pedigree data was consistent with a monogenic autosomal recessive pattern inheritance.

**Conclusions:**

The study describes the clinical and pathological findings of lissencephaly with cerebellar hypoplasia in Churra lambs for which an autosomal recessive inheritance was the most likely cause. Histopathological features observed in the cerebral cortex and hippocampus are consistent with a possible failure in neuronal migration during brain development. This report suggests that lissencephaly should be considered in the differential diagnosis of congenital neurological disease in newborn lambs showing weakness, inability to walk and difficulty sucking.

## Background

Neuronal migration disorders represent a group of rare brain malformations which account for a wide range of cortical anomalies, the most severe forms being represented by lissencephalies [1-3]. Lissencephaly or agyria-pachygyria designates a malformation characterized by a simplified convolutional pattern in which there may be no or only a few broad gyri separated by rudimentary primary fissures and sulci; agyria refers to the extreme in which the brain shows a smooth surface over all the cerebral hemispheres [4]. This term was proposed in humans to distinguish the flat brains of lower mammalian species and at present, several cases have been reported and different studies made in order to clarify its origin [1-3,5,6]. Two main forms of lissencephaly have been described: i) classical or type I lissencephaly, characterized by agyria (total loss of gyri) or pachygyria (few and broadened gyri) together with a very thick cortical gray matter layer, normal brainstem and grossly normal cerebellum or mild vermis hypoplasia, and ii) cobblestone or type II lissencephaly, in which affected children have an agyric brain, with a verrucous appearance and a less marked thickening of the cortex than in type I lissencephaly [1,6,7]. Microscopically, in type I lissencephaly the agyric cortex is mostly composed of four coarse layers: a molecular layer, an outer cellular layer, a sparsely cellular layer and a thick inner cellular layer [7,8]. Type II lissencephaly is characterized by a complete disorganization of the cortex, without discernible layering, and by the presence of gliomesenchymal cell proliferations and neuroglial heterotopia in the leptomeninges [8].

More recently, lissencephaly with cerebellar hypoplasia (LCH) has been recognized as a distinct category of brain malformation, consisting of type I or II lissencephaly associated with cerebellar underdevelopment [1,9-11]. Among LCH, six different phenotypes have been described in children, according to the degree and characteristics of cerebellar hypoplasia and the presence of alterations in other areas such as the corpus callosum and the hippocampus [11].

Lissencephaly in humans is a congenital and genetically heterogeneous disease [3,5,12]. Mutations or deletions in different genes have been identified as involved in lissencephaly cases: the *LIS* gene is responsible for the autosomal form of classical lissencephaly [13] and the doublecortin gene (*DCX or X-LIS*) is X-linked [14] whereas homozygous *RELN* gene has been identified in cases of LCH [10]. Recently, mutations in gene *TUBAIA*, coding for alpha I tubulin, have been also described in some cases of LCH [15].

In domestic animals, lissencephaly is an extremely rare brain malformation. Sporadic cases have been described in dogs [16-18], where a breed predisposition for this malformation has been reported in Lhasa Apso dogs [16,17] and more recently in cats, in which lissencephaly was detected together with microencephaly and hypoplasia of cerebellum and corpus callosum [18,19]. The developmental mechanisms responsible for the absence of gyral formation in animals are not known but a genetic basis in many cases is presumed [18].

In sheep, brain malformations, mainly cerebellar hypoplasia, have been widely described as associated with *in utero* viral infections caused by orbiviruses [18], pestiviruses [20] and arboviruses such as Cache Valley [21] or Schmallenberg viruses [22], variably accompanied by arthrogryposis or other anomalies, such as hydrocephalus or porencephaly.

The purpose of the present report is to describe the clinical, pathologic and pedigree analyses carried out in several cases of lissencephaly in newborn lambs from a pedigree Churra dairy sheep flock.

## Results

### Case presentation

The affected lambs were from a dairy flock of approximately 1600 adult pedigree Churra breed sheep, including 35 purebred males. The flock is located in the Valladolid province of Castilla y León region (North-West of Spain) and is managed under a semi-extensive system, where the animals graze the pasture in appropriate weather conditions and are kept indoors during the lambing and lactation periods. On average ~2.500 lambs are born in the flock each year. In 2005, the farmer observed some newborn lambs with signs of weakness, shivering, muscular rigidity and recumbency. The animals were unable to stand, had difficulties in sucking their dams and lacked coordination. A total of 23 affected lambs (approximately 0.92% of the total crop in that lambing season) were examined and diagnosed as affected by the same clinicopathological entity. Additionally, another 19 affected lambs born in subsequent years were included in this study. A total of 11 newborn lambs from the same farm but dying from unrelated diseases (watery mouth disease, hypothermia) were used as unaffected controls, after checking that no malformations were present in the brain.

### Pathological findings

**Figure 1:**
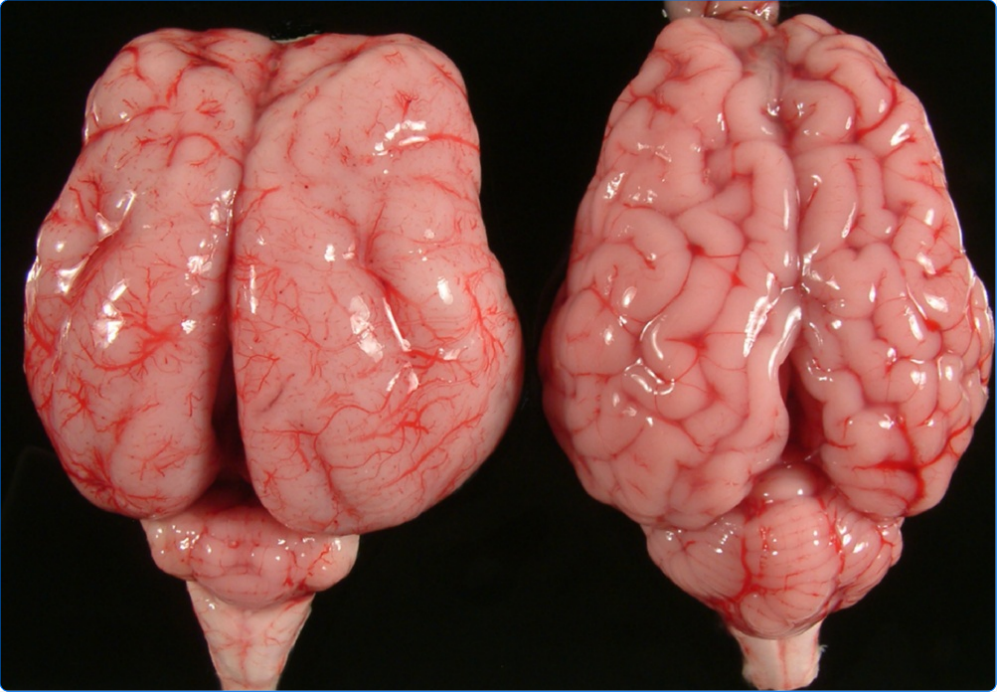
**Affected and normal brains.** Comparative image of a brain showing lissencephaly (left), with few and poorly developed sulci, and a marked cerebellar hypoplasia, and a brain from a newborn control lamb (right).

All the affected lambs showed the same pathological features. A slight reduction in cerebrum size was noted for affected lambs (mean values 5.2 cm length × 6.0 cm width × 3.5 cm height) compared to control lambs (6.4 × 5.7 × 4.3). Agyria was the most remarkable change in the cerebral hemispheres, being particularly evident in the temporal, parietal and occipital lobes, where the cerebral surface had a smooth appearance (Figure 1). Rudimental formation of gyri and sulci were observed in the lateral and rostral areas (Figure 1), probably corresponding to suprasylvian, presylvian and ansate sulci. The cerebellum showed a marked hypoplasia affecting both cerebellar hemispheres and vermis with loss of discernible folial pattern (Figure 1). The mean measurements of the cerebellum of affected lambs (1.4 × 2.7 × 0.8 cm) were clearly reduced compared with unaffected lambs (3.1 × 4.2 × 2 cm).

**Figure 2:**
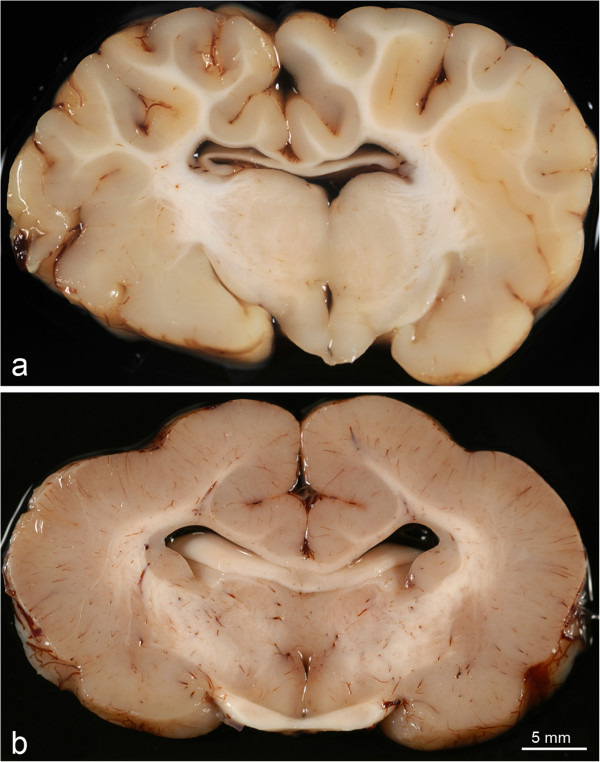
**Coronal section (fixed tissue) of normal (a) and lissencephalic (b) brains.** A marked increase of thickness of the cerebral cortex is seen in the affected lamb **(b)**. Note that the corpus callosum is normally developed.

On coronal sections of the brain, in addition to agyria, a marked pachygyria was observed in all the regions of the cerebral cortex. It was characterized by the presence of a thick grey matter layer, with a depth of 9 mm in average, with broad gyri and very shallow sulci, when present (Figure 2). The white matter was narrow, leading to an inverted grey to white matter ratio. The cerebral grey matter-white matter interface was linear and well defined with rare interdigitations. Macroscopically, the remaining areas of the forebrain (corpus callosum, cingulate gyrus (Figure 2) and hippocampus) and brain stem appeared normally developed. The posterior part of the lateral ventricles was moderately dilated in 13 lambs but no physical obstruction of CSF flow was detected. There was a substantial reduction in the depth of the cerebellar cortex and the folia were markedly decreased in size and number.

**Figure 3:**
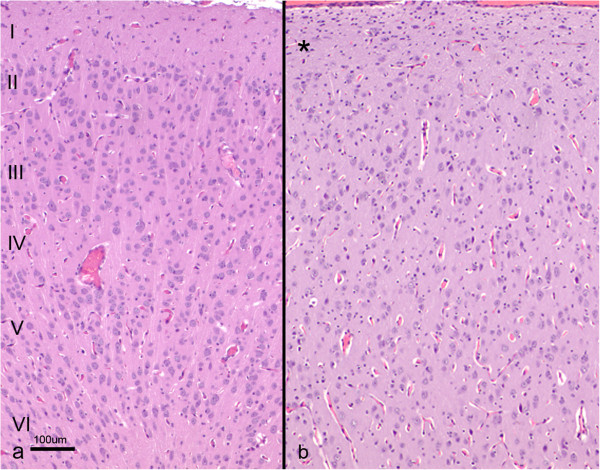
**Histological section of the cerebral cortex from a control (a) and lissencephalic (b) brain.** Both sections were taken at the same magnification. Whereas in the control brain the whole cortex can be seen in the picture and organized in six layers (I to VI), in the lissencephalic brain only the most superficial part of the cortex is shown (aprox. 40% of the whole thickness), due to the thickening of this layer. A sparse-cellular layer underneath the piamater can be identified (*), whereas in the rest of the gray matter the neurons appear disorganized. H-E.

Histopathological examination was carried out in samples from the cerebral cortex of the most severely affected area (temporo-parietal region) in all the animals. The sagittal sections of the cerebral cortex lacked the typical six-layered structure characteristic of this area, seen in the control lambs (Figure 3). Neither glial cells nor neurons were detected in the meningeal layers (Figure 3). In both HE and cresyl violet stained sections, beneath the pial surface, a cell-sparse layer containing neurons smaller than those present in the rest of the cortex and resembling morphologically the molecular or layer I of normal brains, was observed (Figure 3). Below this layer, a wide, more densely populated cellular layer was formed by neuronal bodies of different sizes, some of which had a pyramidal appearance (Figure 3). No laminar organisation or vertical linear array of neurones was identified within this wide layer. The narrow white matter layer was normally myelinated and contained heterotopic neuronal cell bodies.

**Figure 4:**
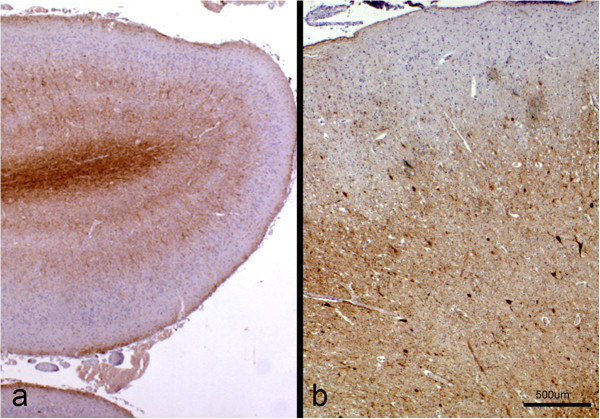
**Immunohistochemistry for neurofilaments.** Sections of the cerebral cortex from a control **(a)** and a lissencephalic **(b)** brain, taken at the same magnification and immunolabelled for neurofilaments. In the control brain, the typical layering of this area (I to VI) can be seen, where the neurons from the pyramidal layers show the strongest signal. In the lissencephalic brain, three different layers can be identified according to neurofilament expression. Immunoperoxidase staining for neurofilaments.

In the control brain sections immunolabelled with antibodies against NF-L neurofilaments, the six conventional layers were identified in the cerebral cortex, and the two pyramidal neurone layers (III and V) had the strongest intensity of immunostaining (Figure 4a). In contrast, in the sections from the affected brains, only three layers were discernible (Figure 4b). Besides the outer marginal zone, corresponding to the molecular layer, that did not show immunolabelling, a thick layer formed mainly by pyramidal neurons of different sizes, some of them showing intense neurofilament expression, was present (Figure 4b). Finally, an internal layer not discernible in HE sections, adjacent to the white matter, with a more reduced immunostaining, was identified (Figure 4b).

**Figure 5:**
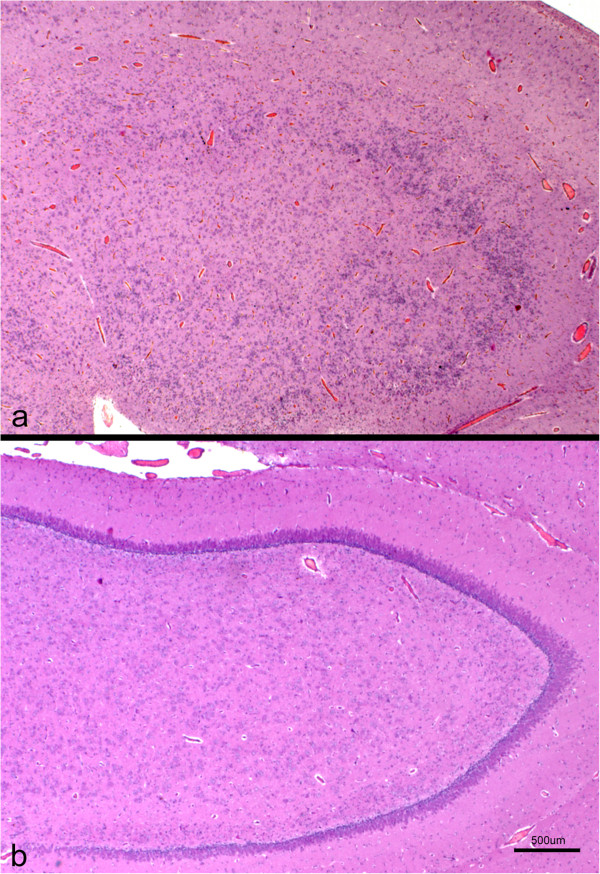
**Cross section of the hippocampus from a lissencephalic (a) and control (b) brain.** Both sections were taken at the same magnification. Whereas in the control brain there is an evident and organized layer of pyramidal neurons, this region shows a marked cellular disorganization in the lissencephalic brain, with several layers of neuronal and glial cells interspersed. H-E.

**Figure 6:**
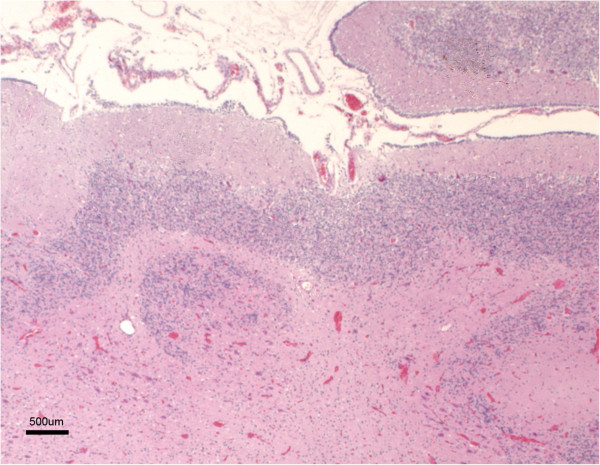
**Marked cerebellar hypoplasia.** The cerebellum shows a reduced number of very short folia. Note that the typical layering of the cortex can be identified. H-E.

**Figure 7:**
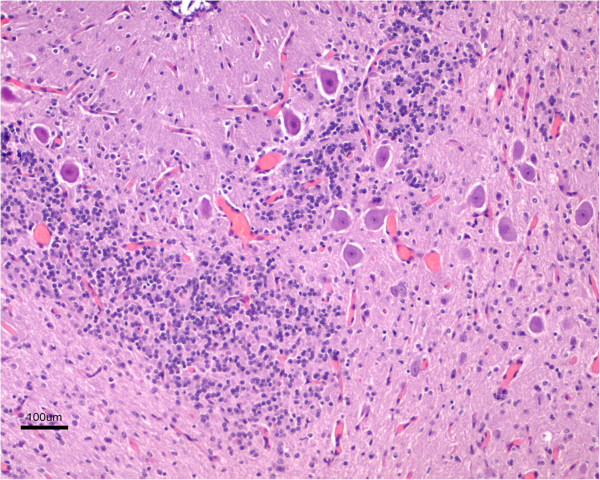
**Cerebellar neuronal heterotopias.** Groups of large neurons, consistent with Purkinje cells, appear in the cerebellar white matter. H-E.

The corpus callosum was present and normally developed in all the lambs, while the hippocampus was disorganized (Figure 5). The pyramidal layer was sharply delineated in the hippocampus of unaffected lambs, whereas in affected lambs several layers of pyramidal neurones were identified interspersed with large numbers of other neurons and glial cells. The dentate gyrus could be discerned in the majority of the lambs, but in some cases it was not clearly evident. The cerebellum of the affected animals showed a marked reduction in size, complexity and number of folia. Lobules were poorly developed and usually the cerebellar cortex was reduced to a single band of folia, which were notably shortened and separated by shallow and wide sulci (Figure 6). Only occasionally up to two folds of folia were seen (Figure 6). The three layers, characteristic of the cerebellar cortex histological architecture, were easily detected in each folium, but Purkinje neuronal heterotopia was commonly seen (Figure 7).

No macroscopic or microscopic changes were detected in any of the other organs of the affected lambs. Pestivirus and bluetongue virus antigens were not detected by immunohistochemical methods.

### Pedigree analysis

**Figure 8:**
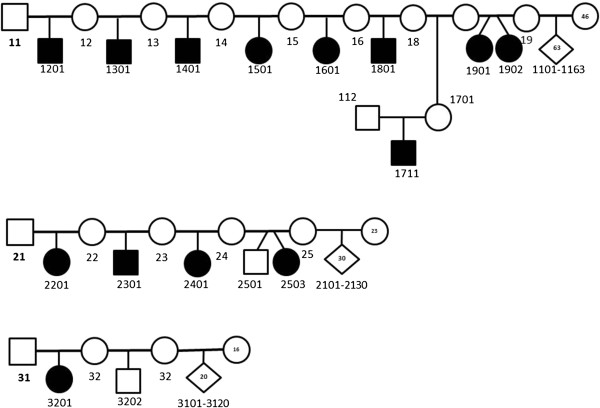
**Pedigree of the lissencephaly and cerebellar hypoplasia affected Churra sheep.** The pattern is compatible with an autosomal recessive inheritance model. The pedigree relationships were confirmed using 19 microsatellite markers. Symbols used in the pedigree are those proposed in humans by the “Pedigree Standardization Work Group of the National Society of Genetic Counselors” [23]. Briefly, square represent male, circle female, shaded square-affected male, shaded circle-affected female. A diamond for the individual symbol is used to indicate animals with gender not specified (males and females). Numbers inside the symbol represent multiple individuals.

The analysis of the three pedigrees, including 14 affected animals, with available DNA, was consistent with a monogenic autosomal recessive inheritance (Figure 8). In the three families some additional healthy lambs were analyzed. Overall, four rams were involved: ram #11 mated 46 ewes that lambed 63 unaffected lambs, 7 ewes that lambed 8 affected labs and one ewe that lambed a non-affected ewe which, when matted to ram #112 produced an affected lamb; ram #21 matted 23 ewes that produced 30 non-affected lambs and 4 ewes that lambed 4 affected and 1 non-affected lambs; ram #31 matted 16 ewes that produced 20 non-affected lamb s and one ewe that produced an affected lamb in one pregnancy and a non-affected lamb in a subsequent parturition.

## Discussion

This study describes forty-two cases of lissencephaly with cerebellar hypoplasia in newborn Churra lambs, occurring over consecutive years, in the same flock. The occurrence of brain malformations of similar type in multiple lambs is not commonly documented in sheep flocks. Most of such cases have been related to *in utero* viral infections by pestiviruses (Border Disease virus), orthobunyaviruses (Schmallenberg, Akabane or Cache valley viruses) or orbiviruses (bluetongue virus) [20-22,24,25]. In our study, evidence of a possible viral etiology of these cases involving the main teratogenic viruses present in our area was not detected by immunohistochemical analyses. Besides, the histological characteristics observed in the cerebral cortex of these lambs are distinct from those occurring in the cerebrum in cases of viral teratogenesis. Porencephaly and hydranencephaly, cerebellar lesions and arthrogryposis are the most common malformations associated with orbiviruses, pestiviruses and orthobunyaviruses respectively. Moreover, immunohistochemical evidence of border disease virus and bluetongue virus was not detected, and the occurrence of these cases predates the emergence of Schmallenberg virus. Although lissencephaly has been reported to occur in association with ovine pestivirus [26] and Cache valley virus [21] infections, the lesions were not described in detail and the lack of gyral formation may have been associated with underlying cortical lesions such as porencephaly. This lesion would therefore differ from the strict definition of lissencephaly. No other cases of lissencephaly were reported from other flocks of Churra or Assaf breed in the area over the same time period. Furthermore, pedigree analysis has demonstrated that lissencephaly cases in this study were compatible with a monogenic trait following an autosomal recessive inheritance, suggesting that lissencephaly is associated with a genetic defect segregating in this flock, rather than a teratogenic infectious disease. This hypothesis has been also proposed in the cases of lissencephaly previously reported in veterinary medicine, that have occurred predominantly in Lhasa Apso dogs [16-18], suggesting a marked breed predisposition. In this study all the cases occurred in purebred Churra lambs, as well as a previously documented case [27] that occurred in the same region but in a different flock with no known direct connection with the flock in this report. However, since many other Churra flocks do not appear to suffer from this condition, it would be premature to hypothesize about a breed-related genetic defect or predisposition.

The exact date of the first occurrence of lissencephaly in this flock is not known. It was not until the farmer noticed that a significant number of animals suffered the disease that veterinary assistance was sought. Given the number of cases in the first year we can make a rough estimate of the frequency of the putative allele causing the disease in the flock. Assuming that the population is in H-W equilibrium, with 23 affected lambs in a total of 2500 lambs born, the allele frequency for the mutated allele in the flock would be approximately 0.09. Since no new rams from other farms have joined the flock in recent years, the most likely reason for this high frequency for a lethal allele will be inbreeding leading to an increased proportion of the flock being carriers of the putative autosomal recessive trait and hence increase the likelihood of mating of carriers leading to birth of affected lambs. Furthermore it should be noted that this problem has not been detected in any other flock belonging to the Churra breeders association. It is also important to note that none of the rams used in the artificial insemination program carried out by the Churra selection scheme has noticed to have any offspring affected by lissencephaly.

In humans, although it is considered a rare disorder, lissencephaly is a well-recognized entity that belongs to the group of malformations caused by abnormal neuronal migration, with two main pathological syndromes described: type I (classical) and type II (cobblestone) [1,7,8]. According to the gross (absence of a verrucous appearance of the cortical surface) and microscopic findings, with no leptomeningeal neuronal heterotopia, the lissencephaly cases recorded in this study resemble the so-called type I or classical lissencephaly of humans. However, the constant presence of cerebellar hypoplasia in these lambs, is consistent with the more recently recognized category of human lissencephaly associated with cerebellar underdevelopment [LCH; 1,9,11], in which an heterogeneity of clinical presentation and imaging findings have been reported [11,15,28,29], probably associated with different gene mutations responsible for malformations in cerebral and cerebellar cortex [1,11]. Ross et al. [11] have proposed a classification of LCH based on clinical and morphological features and six types, so-called LCH groups a-f, have been recognized and some of them related to mutation in some genes. The cases recorded in this study, according to their pathological features, especially the presence of pachygyria, slight microencephaly, a marked thickening of the cerebral cortex, an abnormal hippocampus configuration and a normally developed corpus callosum, most closely resemble the LCH-b group. In humans, this phenotype has been related to mutations in the *RELN* gene that encodes reelin [10,11], a protein secreted by the first born neurons in neocortex and cerebellum [30]. Reelin is an extracellular matrix protein that plays a key role in the organization of architectonic pattern in cerebral and cerebellar cortices, particularly in the radial cortical organization [31] and controlling cell to cell interactions, critical for cell positioning in the brain [2]. Furthermore, lesions found in this study are in accordance with the phenotypic characteristics of the reeler mice, a mouse strain that have a mutation in the *RELN* gene causing a loss of reelin associated with an abnormal neurogenesis in the brain cortex [32]. In mice, mutations in genes that take part of the reelin signaling pathway like mutations in Disabled 1 (Dab1) and double mutations in two lipoprotein receptors, very-low-density lipoprotein receptor (Vldlr) and apolipoprotein E receptor (ApoER2) generate a similar phenotype that is known as reeler-like phenotype [31,33-35]. More recently, a mutation in the *TUBA1A* gene, encoding for the alpha I tubulin protein, has been found in cases of LCH [15,36]. These mutations cause defective interactions in the tubulin heterodimer assembly or in the three-dimensional conformation that will impair the action of these proteins, necessary for central nervous system development [37,38]. Although mutations in this gene result in a variety of phenotypical presentations [15], hypoplasia or complete absence of the corpus callosum, has been reported together with lissencephaly and cerebellar hypoplasia, in the majority of the cases [15,29], including one reported in a cat [19] but was not detected in the lambs in this study. Further studies are necessary to investigate the possible gene mutations involved in this outbreak of lissencephaly in lambs.

Children suffering lissencephaly show mental and psychomotor retardation, seizures that are refractory to treatment and hypertonus in the limbs [7,8]. These clinical signs are broadly consistent with those observed in these lambs, which were unable to walk, had difficulty sucking their dams and show shivering and muscular rigidity.

The gross and, particularly, the histopathological findings of this study consisting of an abnormal layering of the cerebral and cerebellar cortices and the hippocampus, together with the presence of heterotopic neurons in the white matter, are in agreement with a neuronal migration defect during brain development [1,2,6,12]. In classical lissencephaly in humans, four layers are recognized in most of the cases [2,8,36] whereas in LCH a wide variation has been reported, from patients in which no layers can be determined to other showing three or four layers [15,19,28]. In our study, the posterior area of the brain, the most severely affected macroscopically, showed markedly abnormal microscopic architecture. Whereas conventional methods identified two layers, the most external one corresponding to the molecular layer, by using immunohistochemical labelling for neurofilaments, three zones were distinguished. The widest layer was formed by positively immunolabelled neurons, consistent with the pyramidal layers seen in the control brains that also show a strong immunoreactivity. This picture suggests that the most external layer -the molecular area- was formed, and the subsequent migration of the neurons has failed. This finding is consistent with a reeler-like phenotype since the reelin signaling pathway plays a critical role in neuronal migration [39]. A complete and more detailed study of the lesions present in the different areas of the brain in these cases of lissencephaly with cerebellar hypoplasia and the analysis of the genetic basis of the phenotype is necessary to further characterize this entity, and is currently under progress.

## Conclusions

This study reports the clinical and pathological findings of several cases of lissencephaly with cerebellar hypoplasia in pedigree Churra lambs, in which evidence for an autosomal recessive mode of inheritance was demonstrated. The histopathological features observed in the cerebral cortex hippocampus and cerebellum are consistent with a failure in neuronal migration during brain development. Lissencephaly should be considered as a possible cause when newborn lambs show weakness and inability to walk or to suck. Further studies are needed to better characterize this disease in lambs. The possible use of lissencephalic lambs as model for studying human lissencephalies should be also considered.

## Methods

### Pathological examination

Thirty-three out of the 53 lambs examined were submitted alive. After intravenous injection of a veterinary euthanasia drug (T-61, Intervet), a complete necropsy was performed in all the lambs. All the organs were examined and gross lesions recorded. Coronal sections of the brain were made at different levels. Samples from several areas of the brain (cerebral frontal, parietal, occipital and temporal cortex, thalamus and corpus callosum, mesencephalon, hipocamppus, pons and cerebellar peduncles, cerebellar cortex and medulla oblongata) and spinal cord together with other tissue samples (liver, lungs, heart, kidneys, thymus, lymph nodes and intestine with Peyer’s patches) were taken. The tissue samples were fixed in 10%neutral buffered formalin, dehydrated through graded alcohols and embedded in paraffin wax. Sections (4 μm) were stained with haematoxylin and eosin (HE) and cresyl violet method for identification of the neuron structure in the brain.

### Immunohistochemistry

Selected sections from the brain cortex were immunohistochemically labelled with a monoclonal antibody specific for the 70 kDa subunit of the NF-L human neurofilament (clone 2 F11, Dako, Golstrupp, Denmark), at a dilution 1:100, to further characterize brain morphology. Additionally, to rule out the teratogenic infectious agents present in Spain at the moment in which this study was carried out, in selected sections from the brain, thymus, mesenteric lymph nodes and Peyer’s patches obtained from eight affected lambs, immunohistochemistry was applied, using monoclonal antibodies directed against ruminant pestivirus, known to react with border disease virus (PA0801, AHVLA, Addlestone, UK), and bluetongue virus (clone 2E9, Ingenasa, Madrid, Spain) diluted 1:100 and 1:20 respectively. In all the cases, a polymer-based detection system (EnVision + System Labelled Polymer-HRP antimouse; Dako, Golstrupp, Denmark) was employed, following the manufacturer instructions. Subsequently, immunolabelling was developed with a solution of 3,3′diaminobenzidine (DAB) (Vector Laboratories, Burlingame, USA). The slides were counterstained with haematoxylin and mounted. Technique specificity was controlled by omission of the primary antibody and substitution by a normal mouse serum, omission of the Envision polymer and omission of diaminobenzidine. All these controls gave negative results. To evaluate the specificity of the antibodies, tissue samples of a normal lamb brain and several organs of a lamb and sheep previously known to be infected with border disease (where positive signal was found in neurons and lymphocytes) and bluetongue (positive labelling was found in macrophages and endothelial cells) viruses respectively, were employed.

### Pedigree data and analysis

To confirm the pedigree information from the affected lambs and their parents we collected blood samples in a 10 ml Venoject tubes with EDTA (Terumo Europe N.V., Leuven, Belgium) of a total of 33 animals from the lissencephaly affected flock. Fourteen of these animals were affected lambs (cases), three animals were unaffected half-sibs of the cases and 17 were healthy parents of the affected offspring, which were classified as obligate carriers. DNA was extracted from blood samples using the salting out procedure previously described [40]. All the samples were analysed for a set of 19 microsatellite markers amplified in a single PCR reaction following the Multiplex-PCR protocol described by [41]. The PCR products were separated by capillary electrophoresis in an automatic ABI3130xl DNA Sequencer (Applied Biosystems). The results of the capillary electrophoresis were analyzed using GeneMapper v.4.0 software (Applied Biosystems). Finally, a segregation analysis was performed in order to estimate the genetic architecture of the trait. We followed the parsimony principle and, after examining the segregation in the pedigree, we proposed the compatible pattern that makes the fewest assumptions.

## Abbreviations

LCH: Lissencephaly with cerebellar hypoplasia; CSF: Cerebrospinal fluid; H-W: Hardy-Weinberg.

## Competing interests

Authors declare that they have no competing interests.

## Authors’ contributions

VP carried out the pathological examination, participated in the sample collection, interpreted the pathological and immunohistochemical analysis, and wrote the manuscript. ASV performed the pedigree data analysis and helped in the sample collection. MF collaborated in the sample collection and carried out the immunohistochemical analysis. JB participated in the pathological studies, interpretation of the results and helped to draft the manuscript. LD contributed to sample collection and to the pathological studies. MCF collaborated in the interpretation of pathological results and sample collection. JJA interpreted the pedigree data analysis and participated in writing the manuscript. All the authors read and approved the final manuscript.
